# Manganese and Aluminium Recovery from Ferromanganese Slag and Al White Dross by a High Temperature Smelting-Reduction Process

**DOI:** 10.3390/ma15020405

**Published:** 2022-01-06

**Authors:** Artur Kudyba, Jafar Safarian

**Affiliations:** 1 Department of Materials Science and Engineering, Norwegian University of Science and Technology (NTNU), Alfred Getz Vei 2, 7034 Trondheim, Norway; jafar.safarian@ntnu.no; 2 Centre of Materials Research, Łukasiewicz Research Network-Krakow Institute of Technology, Zakopiańska 73 Str., 30-418 Kraków, Poland

**Keywords:** aluminothermic reduction, Al dross, FeMn, ferromanganese slag, white dross

## Abstract

The recovery of Mn and Al from two industrial waste of ferromanganese and aluminum production processes was investigated via implementing a high temperature smelting—aluminothermic reduction process. The experiments were carried out with or without CaO flux addition, and two dross qualities. It was observed that the prepared mixtures of the materials yield homogeneous metal and slag products in terms of chemical composition and the distribution of phases. However, the separation of produced metal phase from the slag at elevated temperatures occurs when a higher amount of CaO is added. Viscosity calculations and equilibrium study indicated that the better metal and slag separation is obtained when the produced slag has lower viscosity and lower liquidus. It was found that the process yields Al-Mn-Si alloys, and it is accompanied with complete recovery of Mn, Si and Fe and the unreacted Al in the process. Moreover, the quality of metal product was less dependent on the slightly different dross quality, and the concentration of minor Ca in metal is slightly increased with significant increase of CaO in the slag phase.

## 1. Introduction

Manganese is an essential element in the production of iron and exists in many steel grades and its primary application is for steelmaking. The second more important application is to produce aluminum alloys. Manganese is produced mostly in the form of ferromanganese (FeMn), silicomanganese (SiMn), and in pure form of electrolytic manganese. In 2019, 4.4 million tons of high carbon FeMn, 1.4 million tons of low carbon ferromanganese, 18 million tons of SiMn were produced [1]. The production of ferromanganese is accompanied with the generation of a high MnO-containing slag that is called high-carbon ferromanganese slag (HCFeMn slag) that has 20–45 wt% MnO. This slag can be fed into the SiMn production furnace as a part of the charge to supply the Mn of the SiMn product, and hence valorize the significant among of the Mn in HCFeMn slag [2]. However, In some cases the usage of HCFeMn Slag in SiMn process is not possible or economic and therefore this by-product is discarded/landfilled. Hence, the valorization of FeMn to recover Mn is important.

Aluminum is the most abundant metallic element in the Earth’s crust, and it has many applications in different industries [3]. Primary aluminum is mainly produced from bauxite ore by the well-known Bayer process (alumina extraction) followed by the Hall–Héroult electrolysis for Al extraction from alumina. The secondary aluminum is produced through the recycling of Al scrap and wasted aluminum products [4,5,6]. In 2020, the global production of metallic aluminum was about 65.3 million metric tons [7]. In the production of primary aluminum, due to the exposure of liquid aluminum to oxidizing atmosphere that is present during the process of melting and alloying, a surface oxidation takes place, leading to the formation of a semisolid skin over the molten Al metal, which also hinders further oxidation. This floating skin is called aluminum white dross (AWS) and consists mainly of aluminum oxide, metallic aluminum, magnesium spinel, periclase, and quartz [8,9]. In general, AWD contains 15–80% metallic aluminum, 20–85% aluminum oxide, 5% salts [10,11,12,13,14]. About four million tons of AWD is produced annually [6,15]. Aluminum dross is a potential toxic industrial waste inevitably generated in aluminum smelter plants and therefore the valorization of Al dross is crucially important.

The treatment of AWD with the aim of metallic Al recovery has been studied through several studies as described previously [16,17,18,19,20,21,22,23,24,25,26,27,28,29,30,31,32]. To establish salt-free processes for AWD valorization and Al recovery, the mechanical treatment was a method that showed feasibility and it was indicated that it is possible to separate most non-metallic components of the AWD in a fine portion via ball milling process [16]. On the other hand, it was shown that pure metallic Al and the Al-rich particles separated from a AWD by mechanical and sizing technique have the potential to be used to reduce MnO-containing slags [17]. However, In the present study the aluminothermic reduction of an industrial HCFeMn Slag by separated Al-containing particles from an industrial AWD is studied in more details to outline an overview about the thermochemistry of the process for valuable metals recovery. The valorization of ferromanganese slag is important as some ferromanganese producers do not valorize it in other processes, and as ferromanganese slags contain usually above 20 wt% MnO, valuable Mn metal is lost. Hence, the novelty of the present work is to show the valorization of two industrial waste to recover Mn and Al metals.

## 2. Experimental Procedure

### 2.1. Material Preparation

Aluminum dross samples were collected from skimmed dross over the surface of molten primary Al and an Al-Si-Mn alloy (grade series 1000 and 4000, respectively) at Hydro. Al dross was subjected to a mechanical milling at room temperature utilizing a ball mill to separate the fine oxide and inclusions of the dross and obtain rich metallic Al-containing particles as applied previously [16,17]. After the mechanical treatment, the milled Al dross was sized by sieving and a particles size ≤ 1 mm for experiments 1, 2 and 1.25–2 mm for experiments 3, 4 was further used. The main differences between the two dross samples were qualitatively studied previously [18], and in Dross 1 there is more Al_2_O_3_, SiO_2_ and and AlN inclusions compare to Dross 2, and Dross 2 has higher content of metallic Al.

**Figure 1:**
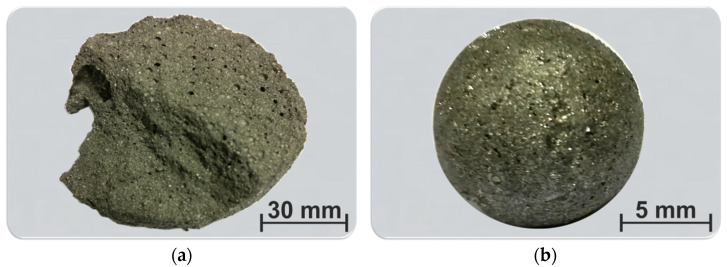
The macro views of samples after experiments: (**a**) a solid slag-metal lump in Exp. 1; (**b**) separated large metal in Exp. 2.

Industrial HCFeMn slag received from the industry was used in this study. In exp. 1, Al dross with the particle size ≤ 1 mm was mixed with the HCFeMn slag. This slag contained 46.5%MnO, 19.7%SiO_2_, 13.9%CaO, 10.9%Al_2_O_3_, 4.1%MgO, 2.3%Fe_2_O_3_, 1.0%K_2_O, 0.9%BaO, 0.4%Na_2_O and 0.3%TiO_2_. In experiment 3, Al dross with the particles size ≤1 mm was used, while the dross with 1.25–2 mm for exps. 2 and 4 was used. Lime flux, CaO with above 99% purity level was in addition with about 10 and 20% of the total masses in experiments 2–4. The charge mixtures details are given in Table 1. The mixtures were charged into alumna crucibles, and then they were put in graphite crucibles as schematically shown in Figure 1a. To prevent graphite contact with the slag and any carbothermic reduction alumina crucible was used, while the graphite crucible was used to get proper induction and heating of the materials.

### 2.2. Smelting-Reduction Tests

The crucibles with the charge mixtures were put in an induction furnace and a thermocouple was put into the charge mixture to measure temperature in the crucible. Each crucible was heated with about 293 K/min to 1823 K and hold for 30 min in a protective atmosphere of Ar with 99.999% purity at const. pressure 1030 mbar. The temperature recordings indicated the reaction kinetics, and it was observed that upon reaching the target temperature (1823 K) for a few minutes, the temperature in the crucible was rapidly increased to a maximum in the range of 1973–2073 K, indicating that the aluminothermic reaction was rapidly taking place, and the temperature inside the crucible declined to the target temperature. Then the furnace power was turned off and consequently the molten components solidified.

The produced metal and slag were separated after breaking the crucibles, and metallography samples were produced from them via mounting in epoxy resin followed by grinding and metallographic polishing. The microstructures and characteristics of these samples were studied by the Zeiss Ultra 55 Scanning Electron Microscope (SEM, Carl Zeiss Microscopy GmbH, Jena, Germany), coupled with energy-dispersive X-ray spectroscopy (EDS). Moreover, the phase analysis of slag samples was studied by X-ray Diffraction (XRD) analysis using Bruker D8 A25 DaVinci X-ray Diffractometer with CuKα radiation with LynxEye™ SuperSpeed Detector (Bruker Corporation, Billerica, MA 01821, USA) [33,34]. The commercial DIFFRAC.EVA software was used to identify the minerals, and quantitative phase analysis of the slags was carried out by TOPAS software.

## 3. Results

### 3.1. Metal and Slag Separation

The visual observation of the products of the Exp. 1 indicated that there are not visible separated metal and slag particles after the high temperature synthesis. However, macroscopic investigation revealed that there are many tiny shining metallic particles in an oxide (slag) matrix (Figure 1a). For the other experiments, it was observed that the metal is under of the slag phase so that large metal phase could be separated as shown in Figure 1b for Exp. 2. In these experiments, the metal had a large single ball shape, such as in Figure 1b, and this metal did not wet the alumina crucible bottom, while was surrounded mostly by the slag. For the Exp. 2 to 4 the separation of the bulk metal and slag was very good, and there was not significant sticking between the metal and slag, and between the metal and crucible.

**Figure 2:**
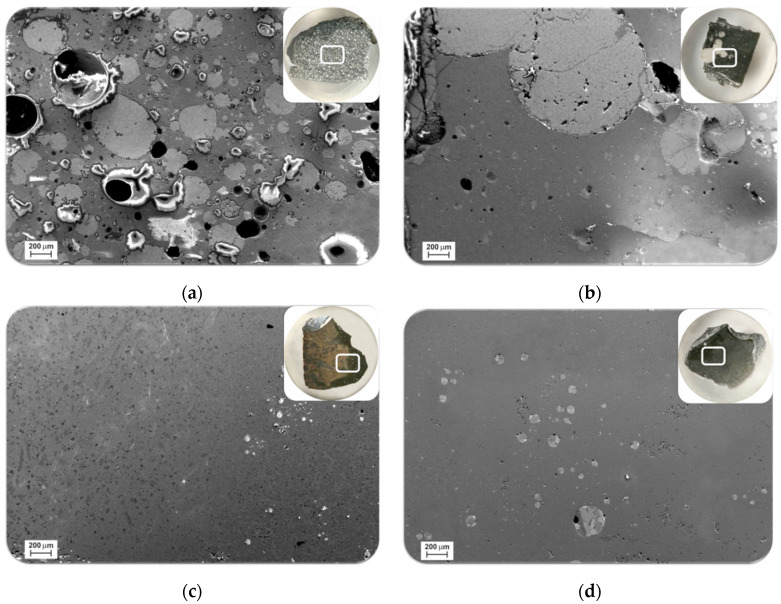
Microstructure of the slags from experiments: 1 (**a**); 2 (**b**); 3 (**c**) and 4 (**d**).

The SEM microstructural analysis of the sample of Exp. 1 showed that there are many spherical metallic droplets in a slag matrix (Figure 2a). Inspecting the metallic particles over a large area indicated that these metallic particles are mostly smaller than 300 μm. For the Exp. 2 with dross 2 and the addition of 10% CaO we observe that the separation of the metal and slag is much better; however, there is still significant small metallic particles in the slag matrix. Figure 2b clearly shows the metallic product in this sample is mainly in the large drops; however, there are many tiny metallic droplets that are distributed all over the slag matrix. Inspecting a wide area of the sample indicated that the majority of these solidified metal droplets are smaller than 100 microns. The microstructures of slags produced from Exp. 3 and 4 (Figure 2c,d) show that there are fewer metallic droplets in the slag matrix than Exp. 2.

### 3.2. Characteristics of Produced Metals

#### 3.2.1. Metal Composition

The measured overall compositions of the metal products are given in the Table 2. Making EDS point and area analysis of many metal droplets in each sample indicated that they have the same chemical composition. Hence, the information in Table 2 is representative of the whole metal product. It was found that for all the experiments the Mn content of the metal is in the range of 31–32.4% and its origin is mainly the HCFeMn slag. The other main metallic components were Al (40–42%) and Si (21–22%), and their origin is the AWD. The concentration of Fe is low in all metallic products (2.6–3.4%) and its main source is the HCFeMn slag, which is low in Fe oxide. The concentration of Ca in the metallic product of Exp. 1 is insignificant compared to the Exps. 2 to 4. Obviously, there is more Ca in metal when more CaO is added to the reactants.

#### 3.2.2. Microstructural Analysis

**Figure 3:**
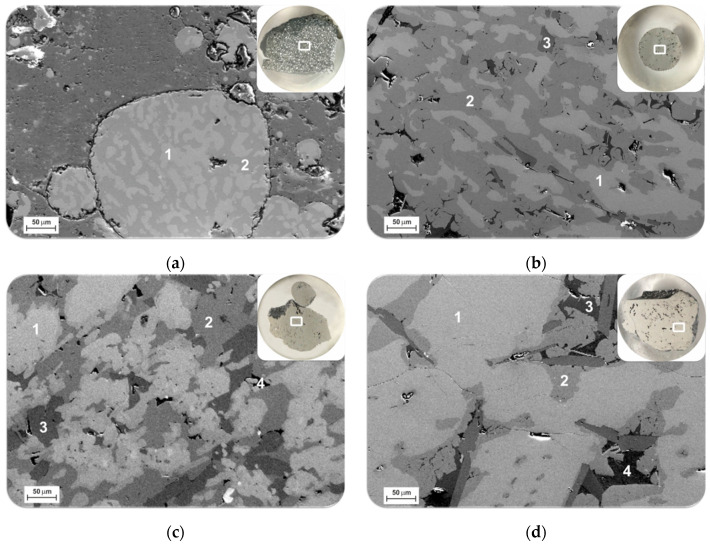
Backscattered SEM images at magnification 500× of the produced metals in experiments: 1 (**a**); 2 (**b**); 3 (**c**); 4 (**d**).

The microstructural analysis of the produced metals indicated that Metal 1 (from exp. 1) has two main phases as identified by points 1 and 2 in image (a) in Figure 3; a bright phase 1, and dark grey matrix 2. Similarly, in Metal 2 there is a main bright phase 1 that is distributed in the matrix phase 2 (Figure 3b). In addition, two darker phases 3 and 4 are observed. In metals 3 and 4 (from Exp. 3 and 4, respectively) the brightest phase 1 is the dominant phase, while the order of other phases are the grey phases 2, grey phase 3 and the dark phase 4 (Figure 3c,d).

The compositions of the observed phases in metal products were studied by point analysis as given in Table 3. Obviously, the brightest phase 1 has the highest concentration of Mn and Fe, while the darkest phase 4 has the highest concentration of Si. The observed different microstructures for the produced metals compared to their close overall compositions are due to the distribution of the phases, which are different. In Metal 1 a fine structure of the phases 1 and 2 is observed, while the size of the phases 1 and 2 in Metals 3 and 4 are larger.

### 3.3. Characteristics of Produced Slags

#### 3.3.1. Microstructural Analysis

The measured overall compositions of the slags are given in Table 4. The provided compositions are the averages of 4–6 EDS measurements in large areas with further conversion of elements to more stable forms of their oxides. It is worth to note that the obtained EDS analysis results for different positions in each slag sample were very close that indicates the measurements are reliable and the slags are homogeneous in microstructure regarding the distribution of the phases.

**Figure 4:**
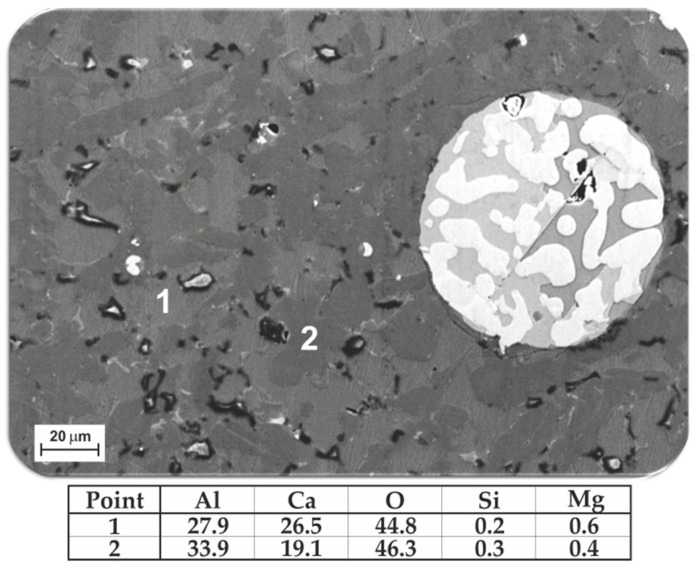
Microstructure of slag of Exp. 3 with the measured composition of the main phases (at.%).

The microstructural analysis by SEM for the slags indicated that the produced slags have different calcium-aluminate and calcium-aluminosilicates, depending on the overall composition. Figure 4 shows typical SEM image and the measured chemical compositions of the dominant phases in Slag 3 (produced in experiment 3). As seen, the slag has two main phases that are rich in Al, Ca and O. The other phases were quite fine, and it was not possible to obtain precise EDS point analysis, however, they contained mostly Si, Al, Ca, O, C and N elements, and C and N were qualitatively higher than the areas around. Therefore, further XRD analysis was performed to identify the phases in the slags.

The SEM study of slags 1 and 3 indicated that they contain some tiny dark particles as seen in images of Figure 3a and Figure 4, while such particles were much less in slags 2 and 4. These particles could not be characterized due to their small size, and they are probably carbides and nitrides particles that are in significantly higher content in Dross 1 compared to Dross 2 as characterized previously [17].

#### 3.3.2. Phase Analysis of Slag

**Figure 5:**
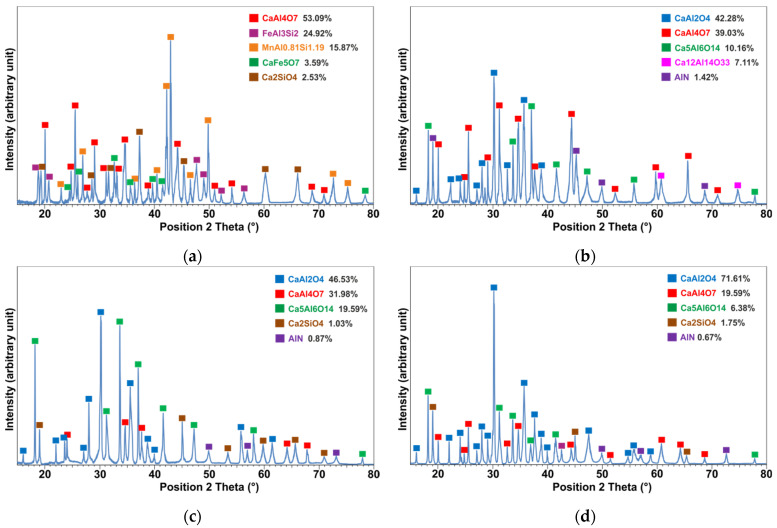
XRD patterns of the produced slags in experiments: 1 (**a**); 2 (**b**); 3 (**c**); 4 (**d**) and semiquantitative identified phases.

As mentioned above, the separation of the metal and slag phases in the Exp. 1 was not possible, and therefore a large portion of the product was milled and analyzed by XRD. Figure 5a indicates that the produced metal has Mn, Al, Si and Fe as the main elements. Two metallic phases could be identified as Mn_5_Al_4_Si_6_ and FeAl_3_Si_2_ using the XRD database. The Mn_5_Al_4_Si_6_ intermetallic phase is not found in the Al-Mn-Si phase diagram [35] and does not fit well with the EDS analysis of the dominant phase in the EDS analysis. The closest composition to this compound in Al-Mn-Si system is Al_3_Mn_3_Si_4_ that usually comprises 37–27 at.% Al and 30–40 at.% Si at constant Mn content of 33 at.%. The other identified FeAl_3_Si_2_ phase by XRD was not found by SEM microstructural analysis as a main phase (Table 4). Figure 5a indicates that the produced slag in Exp. 1 consists mainly of monocalcium di-aluminate (CaO·2Al_2_O_3_) and minor oxides of magnesium-aluminate and calcium ferrite.

The produced slag in Exp. 2 has different kind of calcium aluminates of CaO·Al_2_O_3_, 5CaO·3Al_2_O_3_, CaO·2Al_2_O_3_, 12CaO·7Al_2_O_3_, which are called for simplicity hereafter CA, C_5_A_3_, CA_2_, C_12_A_7_, respectively (Figure 5b). The slag has minor amount of aluminum nitride (AlN) phase. Overall, the obtained slag in Exp. 2 has more than 98% calcium aluminate phases and CA_2_ is the second dominant phase with about 39% according to the Topas analysis, which is less than the amount of CA_2_ in the slag of Exp. 1. The main phases in the slags of Exps. 3 and 4 (Figure 5c,d) are similar to Exp. 2 (Figure 5b); however, the amount of CA_2_ in Exp. 3 is more than that in Exp. 4.

## 4. Discussion

The above obtained results are discussed as follows.

### 4.1. Physiochemical Properties of the System

The produced metals and slags in all experiments have obtained a molten state and then solidified. This is corroborated regarding the homogeneous microstructures of the metal and slag phases and the shape of the metallic phase (spherical droplets) and the proper contact of the metal and slag observed in Figure 2, Figure 3 and Figure 4. In principle, the separation of metal and slag has been occurred due to the density differences. Considering the densities of the metallic components in molten state and making a fair approximation of no significant volume change due to mixing, for the produced alloys with compositions in Table 2, we obtain densities of 3.7, 3.6, 3.7 and 3.7 g/cm^3^ for alloys 1 to 4, respectively. The densities of the slag compositions 1 to 4 can be estimated by the available data in literature as 2.6 for the ternary CaO-Al_2_O_3_-SiO_2_ slag 1, and 2.73, 2.72 and 2.73 for the binary slags 3 to 4, respectively [36]. Obviously, the significant differences between the densities of the metal and slags in each experiment could provide proper slag and metal separation as expected. However, not good separation of the metal and slag phases in Exp. 1 is attributed to the higher viscosity of the slag 1.

**Figure 6:**
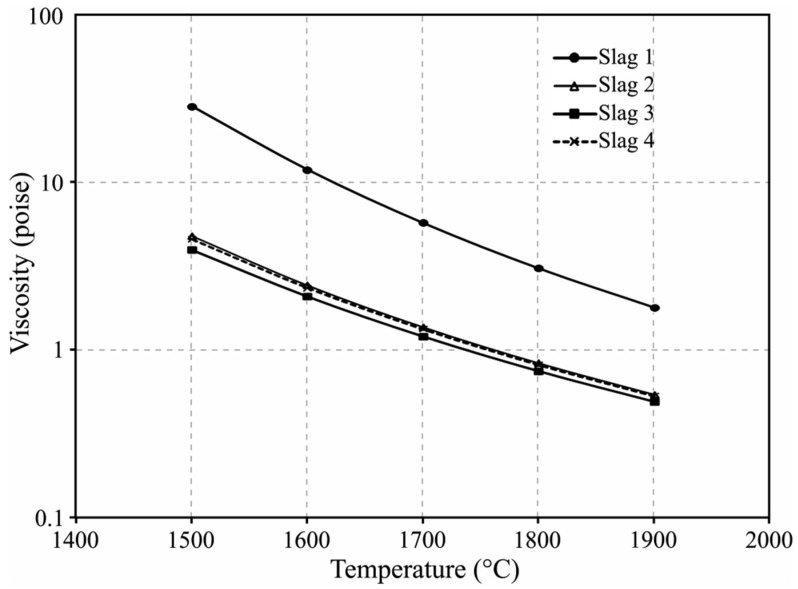
Calculated viscosities for slags 1 to 4 at elevated temperatures.

To evaluate the effect of viscosity on the separation of metal and slag, the viscosities of slags 1 to 4 were calculated by the viscosity module of FactSage software, version 8.1. Figure 6 shows the results for the temperature range of 1500–1900 °C, the temperature range that the materials experienced during holding at target temperature and the aluminothermic reduction step. It is observed that the viscosities of slags 2 to 4 are 6–7 times lower than slag 1. Therefore, we may conclude that in the Exp. 1 there has been no proper accretion of the molten metal droplets in a relatively viscose slag to form large metal particles that could sink to the crucible bottom (Figure 3a). Whereas, in Exps. 2 to 4 the low viscosity of the slags could provide proper conditions for the metal droplets accretion parallel to sinking to the crucible bottom.

It was mentioned above that slags 1 and 3 contained some inclusions such as AlN and they could provide higher real viscosities than the calculated values for these slags in Figure 6. Obviously, for experiment 3 with significant CaO addition, the effect of these particles was less than the slag 1 without CaO addition. Hence, the better separation of metal and slag in Exp. 3 compared to Exp. 1 is attributed to the significantly lower overall viscosity even with the existence of solid particles.

### 4.2. Evaluation of Aluminothermic Reduction

The chemical compositions of the produced metal and slag phases indicate that significant mass transport has been occurred through the contact of Al with the ferromanganese slag and lime oxide mixture. The melting point of the Al (in dross) and the used ferromanganese slag are 660 °C and 1200–1300 °C, respectively. The latter was observed in the experiments upon heating and is confirmed by the liquidus in MnO-SiO_2_-CaO-(Al_2_O_3_) phase diagrams [36]. Hence, at the target temperature of 1500 °C, the two reactants are achieving a molten state. In the Al dross, the metallic particles have usually an oxide skin (Al_2_O_3_), and this oxide is rapidly dissolved into the adjacent FeMn slag when this slag is getting molten state. For the experiments 2 to 4 that the powders of CaO and FeMn slag were mixed before adding into the crucible, CaO dissolution into the slag occurs rapidly when the HCFeMn slag starts to melt and if the aluminothermic reduction reactions occur and temperature rises, the dissolution of CaO occurs faster. The observation of no free Al_2_O_3_ and CaO particles in SEM and XRD analysis after the reactions and obtaining homogeneous overall slag compositions confirm the complete dissolution of Al_2_O_3_ and CaO components in the charged materials. When the aluminothermic reduction occurs, the mass transport of the Al, Mn, Fe, Si, Ca and Mg between the two molten phases occurs via the following reactions and they led to the obtained metal compositions in Table 2.

3MnO + 2Al = 3Mn + Al_2_O_3_ ΔH°_(1500 °C)_ = −466 kJ/mol. (1)

2Al + Fe_2_O_3_ = 2Fe + Al_2_O_3_ ΔH°_(1500 °C)_ = −877 kJ/mol. (2)

2Al + 1.5SiO_2_ = 1.5Si + Al_2_O_3_ ΔH°_(1500 °C)_ = −262 kJ/mol. (3)

2Al + 3CaO = 3Ca + Al_2_O_3_ ΔH°_(1500 °C)_ = 250 kJ/mol. (4)

2Al + 3MgO = 3Mg + Al_2_O_3_ ΔH°_(1500 °C)_ = 142 kJ/mol. (5)

Reactions (1) to (3) proceed significantly regarding the existence of more metallic Al in the charge than the stoichiometric of these reactions, and as MnO, Fe_2_O_3_ and SiO_2_ are less stable than Al_2_O_3_. SEM and XRD results indicated that complete reduction of Mn and Fe from the molten slag phase occurred and as there is significantly higher amount of Al than the stoichiometric reactions for the reduction of reducible oxides, some unreacted Al is left that exist in the final metal product. It is worth to note that Si exist in the dross in the form of both elemental Si and SiO_2_ as the dross is a complex material [16]. Moreover, some SiO_2_ exist in the slag before the reaction starts. Therefore, the reduction of SiO_2_ in the system and its distribution between the metal and slag products is dependent on the amount of the SiO_2_ in the dross and its form. Obviously, if the elemental Si content in the dross is low and less than the equilibrium concentration in the final metal product, the reduction of SiO_2_ occurs and Si is transferred to the metallic product. However, if the elemental Si in the dross is high and more than the equilibrium concentration in the final metal product, SiO_2_ reduction does not occur and SiO_2_ from the dross gets dissolved into the coexisting slag phase, and in addition Si transfer from the metal happens via a couple of possible reactions such as silicothermic reduction of MnO and FeO in parallel to the main reactions (1) and (2) for Mn and Fe formation. The XRD analysis results indicated that there is no significant SiO_2_ in the slags 2–4 and Si in the produced slags was mostly found in the Ca_2_SiO_3_ minor phase, and the semi-quantitative analysis indicates that the amount of Si in the slag is low and hence Si transfer between the two phases has been mostly via chemical reaction (3). The existence of more SiO_2_ in slag 1 than the other slags may be due to not proper process time for the complete reduction of SiO_2_ from slag 1. Obviously, the SiO_2_ concentration in slag 1 is higher than slags 2–4 and there is a higher SiO_2_ chemical activity and so driving force for the chemical reaction (3) for slag 1 than the other slags. However, the rate of reaction (3) from slag 1 is much slower than that for slags 2–4 due to the slow mass transport of SiO_2_ in the bulk viscose slag 1 to the slag/metal interfacial reaction, which is obviously not a mass transport step for slags 2–4. Hence, the SiO_2_ reduction rate is controlled by the mass transport in the slag phase and the applied temperature and duration has not been enough for the completion of reaction (3) in Exp. 1.

Parallel to significant Mn, Fe, Si reduction, there is partial reduction of CaO and MgO in the system and it caused small amount of Ca and Mg transfer into the molten metal phase. The higher, Ca concentration in Exps. 3 and 4 compared to Exps. 1 and 2 indicates that when CaO content of slag is higher more CaO is reduced. This is due to the higher chemical activity of CaO in the slag and larger driving force for chemical reaction (4) to proceed in a reaction time. The concentration of Mg in metal products is close and obviously there is insignificant MgO reduction, which is due to its low concentration in all slags with low chemical activity.

### 4.3. Distribution of Elements and Equilibrium Study

**Figure 7:**
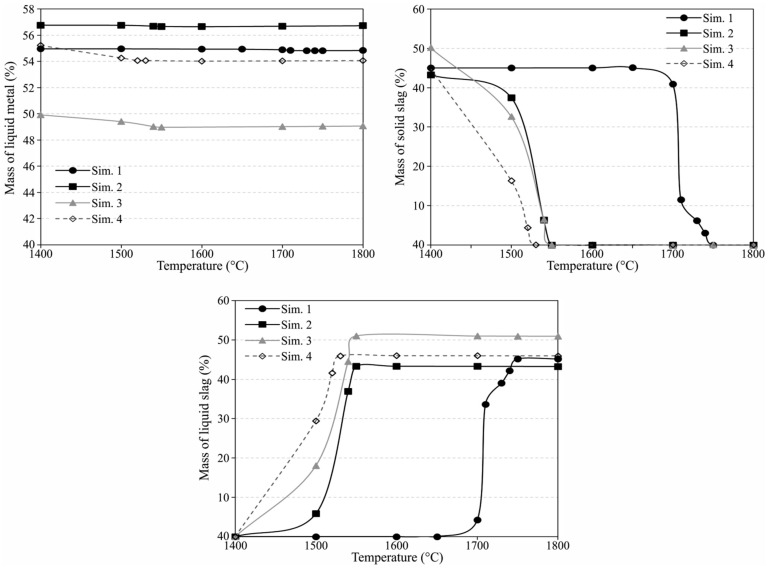
Mass fractions of the coexisting phases in Sim.1 to 4 per unit mass of the charge mixture.

To study the distribution of elements between the slag and metal phases, the process was simulated considering the main components of the utilized FeMn slag (MnO, SiO_2_, CaO, Al_2_O_3_, MgO, Fe_2_O_3_), and considering the dross 1 has 70%Al, 15%Si and 15%Al_2_O_3_, while the dross 2 has 80%Al, 15%Si and 5% Al_2_O_3_. The considered Al_2_O_3_ contents were typical assumptions based on our previous study [16]. The existence of significant metallic Si in the drosses is also based on the above obtained compositions of the produced metals and slags. The equilibrium study was carried out using FactSage thermodynamic software version 8.1, and applying FactPS, FToxide and FTlite dtabases. The calculations were for the temperature range of 1400 to 1800 °C that the metal and slag products are formed, and the results are discussed as follows. These calculations by FactSage hereinafter are called Sim. 1 to Sim 4 corresponding to Exp. 1 to Exp. 4, respectively. The mass ratios and mixing of the above-mentioned dross 1 and 2 with HCFeMn slag were according to experiments 1 to 4 in Table 1. Figure 7 shows the mass of the produced metal and slag products per unit mass of the reactants mixture and as we see the mass of metal and slags products are different. For a given test, the total mass of the metal is not significantly changed with temperature, indicating not significant change of the slag mass and in other word not significant mass transfer of the elements between the slag and metal phases with temperature drop after the completion of reduction reactions. However, Figure 7 shows that the amount of the liquid and solid phases of the slag are changed by temperature, which is expected regarding the solidification upon cooling below the liquidus. Moreover, these changes depend on the charge mixture. The higher portion of metal formed in Sim. 1 and Sim. 2 compared to the Sim. 3 and Sim. 4 is due to the lower CaO addition so that the mass of the metallic product per unit mass of the charge becomes higher. On the other hand, the mass of metal in Sim. 2 is greater than Sim. 1 due to the application of the higher grade of dross 2, which has lower amount of aluminum oxide than dross 1. Similarly, the higher metal production in Sim. 4 compared to Sim. 1 can be explained.

#### 4.3.1. Characteristics of Slag

In the aluminothermic reduction temperature of the material mixture rises (above 1700 °C) due to the exothermic reactions and reactions (1) to (5) are almost completed for experiments 2–4. Therefore, the molten slag and metal products coexist for a while at elevated temperatures. Figure 7 indicates that the solidus of slag in Sim. 1 is about 1650 °C, and the calculations showed that the slag contains solid particles of CaMg_2_Al_16_O_27_ above this temperature up to the liquidus at 1740 °C. Hence, it is expected that the slag is more viscose for Exp. 1 than Exps. 2 to 4 and therefore not proper separation of slag and metal occurs. This explanation here is corroborating the above discussed effect of viscosity on metal and slag separation. However, for the viscosity calculations for Figure 6 the minor components in the slags were considered and the results in Figure 6 for Exp. 1 are reliable and must be lower than any viscosity that FactSage predicts for Sim. 1 slag. The FactSage calculations indicated that for Sim. 1 to Sim. 4 there are three stable phases of CaAl_2_O_4_, Ca_3_MgAl_4_O_10_ and Ca_2_Mg_2_Al_28_O_46_ at room temperature and their amount is changing with the slag compositions changes. These are different with the XRD analysis results, and the latter two phases are negligible with regard to the small amount of MgO in the charge, and hence the observed calcium aluminate species in Section 3.3.2 are more reliable to consider.

#### 4.3.2. Characteristic of the Metal

**Figure 8:**
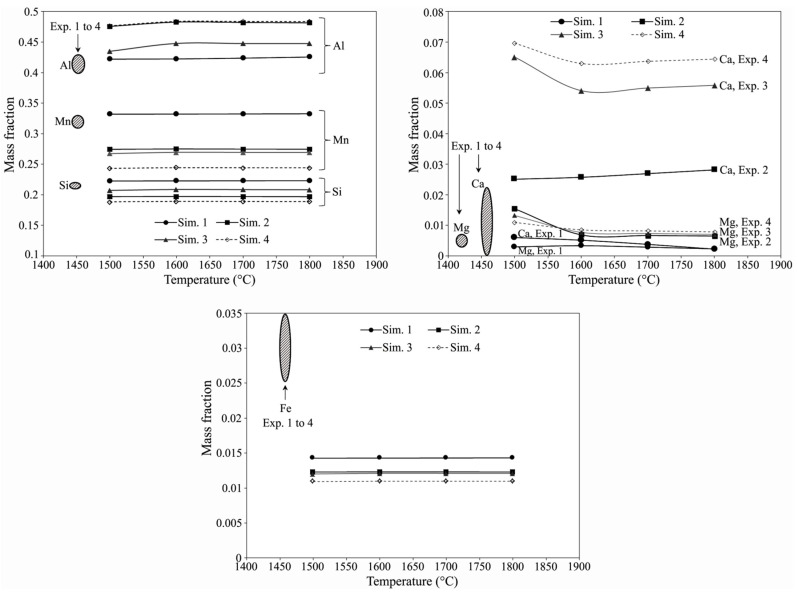
Calculated metal compositions in Sim. 1 to 4 and the measured metal compositions in Exp. 1 to 4.

The FactSage calculations showed that the liquidus of metals in Sim. 1 to 4 are 950, 960, 1000, 1040 °C, respectively. However, the solidus for the alloys is very close and between 780–800 °C. Hence, the produced metals in the experiments were molten at much lower temperatures than the solidus of the slags, which were above 1400 °C (Figure 7). As the metal products are surrounded by the slag (with low thermal conductivity), temperature of the metal products is decreased slowly and hence the produced metal microstructures in the experiments are representative of the phases that may form under equilibrium cooling. FactSage calculations revealed that the produced metals in Sim. 1 to Sim. 4 calculations all have CaAl_2_Si_2_, Mn_11_Si_19_, Al_14_Fe_3_Si_3_, Al_4_Mn phases at room temperature, which are significantly different with the above observations in Figure 3 and Table 3. The only phase that FactSage predicts and was also observed in SEM is the phase number 3 in Table 3, which is most likely CaA_l2_Si_2_. Regarding these differences and the data in literature mentioned in Section 3.2, we may conclude that the Al-Mn-Si-(Ca-Fe) system must be further studied and assessed, and the related thermodynamics databases be improved. Regarding these, for evaluating the FactSage calculations and comparing them with the experimental results we consider the metals in molten state at elevated temperatures. Figure 8 shows the mass fractions of the produced metals via Sim. 1 to Sim. 4 calculations and their comparison with the measured compositions in Experiments 1 to 4. Obviously, the calculated concentrations of the main elements of Al, Mn and Si are in correlation with the concentrations of these elements in the experiments. This may reveal that the FactSage databases for molten slag-metal studies of this study are more reliable than solid state as was discussed above.

The calculated Al contents in Sim. 1 to Sim. 4 in Figure 8 are close; however, they are higher for Sim. 2 and Sim. 4 compared to Sim. 1 and Sim. 3. This is not in agreement with the corresponding experiments 1 to 4 presented in Table 2. In contrast, Mn content in Sim. 1 is much higher than Sim. 4, while the results for Sim. 2 and Sim. 4 are close. These differences are related to the amount of metallic Al (in dross) introduced into the system and it yielded different levels of Al with respect to Mn in the final alloy. As Mn in the charge is ended up in the metal product, the calculated results in Figure 8 show that the mass of the unreacted metallic Al is the highest for Sim. 4 and Sim. 2, followed by Sim. 3 and Sim. 1, respectively. To better evaluate, the consumed masses of Al for the main reactions (1) to (3) were calculated and then the ratio of the masses of unreacted metallic Al over introduced Mn was calculated. The Al/Mn ratio became roughly 1.3, 1.8. 1.8 and 2.1 for Sim. 1 to Sim. 4, respectively, which agrees with the results in Figure 8. The introduced Si in the charge for all scenarios is close and as the masses of the metals are different, we observe different Si concentrations for different calculations in Figure 8. There may be a correlation between the Si concentration and the mass of the Al, Mn and Fe components in the alloys. Mass balance calculations showed that the mass of Si over the total mass of Al + Mn + Fe in the alloys is slightly different as 0.28, 0.25, 0.27, 0.25 for Sim. 1 to Sim. 4, respectively. This agrees with the obtained results in Figure 8.

Comparing the produced metals in Exp. 1 to 4, similar trend for the Ca concentrations in the metal products is observed in Sim. 1 to Sim 4. Obviously, Ca in metal is higher when more CaO is added to the system as it increases the concentration of CaO in the slag phase and therefore it provides higher chemical activity and hence more transfer of Ca into the metal product via chemical reaction (4) as mentioned previously. Observing higher Ca concentrations in the metal products in Sim. 2 to 4 than the experiments may be attributed to the real lower chemical activity for CaO in the slag phase due to the existence of other components (e.g., K_2_O, TiO_2_, BaO). Figure 8 shows that the calculated Mg concentrations in the metal products are in relatively good agreement with the experimental results and very small amount of Mg is reduced in the system.

### 4.4. Slag Chemistry Optimization

**Figure 9:**
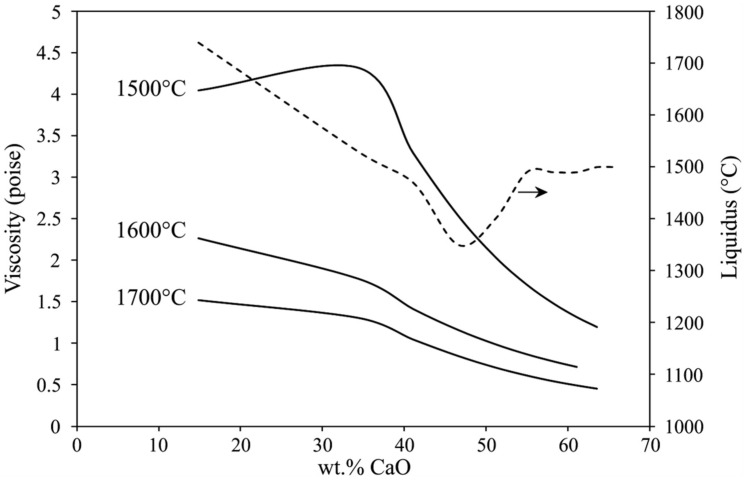
Effect of CaO addition and temperature change on the viscosity (solid curve) and liquidus (dashed-line curve) of slag of Sim. 1.

The experimental and thermodynamic calculations above indicate that the addition of CaO into the reactants is a key parameter that affects the chemical composition of the products and the separation of metal and slag products. Figure 9 shows the results of FactSage calculations about the viscosity and liquidus when CaO is added to the slag of Sim. 1. As we see, CaO addition lowers the slag viscosity and at higher temperatures we obtain lower viscosity. Moreover, the liquidus is lower when CaO content is in a range of 40 to 55 wt%, while the viscosity is lower than lower CaO-containing slags. Therefore, the CaO addition must be controlled to have a final calcium-aluminate slag with this CaO compositional range.

The calcium-aluminate slag that is produced via the presented process can be used for alumina production as outlined previously [18]. According to Azof et al. [34,37,38] the most leachable calcium aluminate phase is maynite (C_12_A_7_) and if the CaO concentration is about 48 wt% or a few weight percentages higher, the produced slag has significant amount of this phase, when SiO_2_ content of slag is low. Hence, if the slag is valorized later for alumina production, the CaO addition must be fixed to attain the highest alumina recovery.

## 5. Conclusions

The interaction of Al white dross with high-carbon ferromanganese slag at elevated temperatures was carried out to recover valuable metallic components of Al, Mn and Si. The main conclusions are summarized as follows:

1. The process provides complete recovery of Mn, Si and Fe from the reactants;
2. Metallic Al in aluminum dross is partially consumed for the aluminothermic reduction reactions, and the rest is completely recovered in the metal product;
3. The overall chemical composition of the metal product is not significantly affected by CaO addition, while the minor Ca concentration (still below 0.1 wt%) is slightly increased with significant CaO addition;
4. The viscosity of produced Al_2_O_3_-containing slag is significantly dependent on the CaO in the slag, and it is decreased with increasing the CaO content, and the viscosity can be optimized to below 2 P with more than 30 wt%CaO in slag;
5. The liquidus of the slag phase is significantly dependent on the amount of CaO flux, and it is possible to obtain fully molten slag phase with adjusting the added CaO between 40–60% above 1500 °C;
6. The separation of molten metal from the coexistence slag phase is better when the slag is less viscose and has lower liquidus temperature.

## Figures and Tables

**Table 1:** The charge mixtures details.

Exp. Number	HCFeMn Slag (wt%)	CaO Addition (wt%)	Al Dross (wt%)
1	50	-	Dross 1 *: 50
2	45	10	Dross 2 **: 45
3	40	20	Dross 1 *: 40
4	40	20	Dross 2 **: 40

*—Dross with a particle size: ≤1 mm. **—Dross with a particle size: 1.25–2 mm.

**Table 2:** Measured overall compositions for produced metals in Exps. 1–4 (wt%).

Experiment Number	Metal Overall Compositions					
	Al	Mn	Si	Mg	Fe	Ca
1	42.7	31.0	22.9	0.4	3.0	0.0
2	42.2	31.8	22.2	0.6	2.6	0.6
3	40.0	32.1	21.9	0.7	3.4	1.9
4	40.4	32.4	22.0	0.4	3.2	1.6

**Table 3:** The measured compositions (wt%) of the phases in metal products.

Phase No.	Al	Si	Mn	Fe	Ca	Mg
1	39–40	14–18	38–42.5	3.2–3.3	<0.02	0.3–0.45
2	32–41	26–37	26–29	1.5–3.5	<0.02	0.3–0.5
3	29–32	37.7–40	0.3–0.9	0.1–0.4	23.5–27.5	0.4–0.6
4	0.6–1.1	96.5–98.6	0.1–0.3	<0.03	<0.03	0.1–0.3

**Table 4:** The overall compositions of the slags (wt%).

Experiment Number	Slag Overall Compositions						
	Al_2_O_3_	SiO_2_	MnO	MgO	FeO	CaO	P_2_O_5_
1	64.6	15.9	0.02	0.56	0.10	18.5	0.32
2	65.3	1.1	0.00	0.82	0.03	32.7	0.05
3	56.2	3.6	0.05	2.4	0.07	37.6	0.08
4	59.9	1.2	0.03	1.5	0.03	37.3	0.04

## Data Availability

Data available in a publicly accessible repository that does not issue DOIs. Publicly available datasets were analyzed in this study. This data can be found here: https://www.ntnu.edu/metpro (accessed on 3 November 2021).
